# Supplementary material for: Stacking Model‐Based Classifiers for Dealing With Multiple Sets of Noisy Labels
Source: Biom J. 2025 Mar 12;67(2):e70042. doi: 10.1002/bimj.70042 (PMC11898607; doi:10.1002/bimj.70042)
Supplement: Supplementary file 1 — Supporting Information [file BIMJ-67-e70042-s001.zip › density-based-ensemble-model-main/competitors_real_data/netlab-master/netlab-master/help/demknn1.htm]

Netlab Reference Manual demknn1


# demknn1

## Purpose

Demonstrate nearest neighbour classifier.

## Synopsis

```
demknn1
```

## Description

The problem consists of data in a two-dimensional space.
The data is
drawn from three spherical Gaussian distributions with priors 0.3,
0.5 and 0.2; centres (2, 3.5), (0, 0) and (0,2); and standard deviations
0.2, 0.5 and 1.0. The first figure contains a
scatter plot of the data. The data is the same as in `demgmm1`.

The second
figure shows the data labelled with the corresponding class given
by the classifier.

## See Also

`dem2ddat`, `demgmm1`, `knn`

---

**Pages:**
Index


---

Copyright (c) Ian T Nabney (1996-9)
